# The Association of a Previous Cesarean Section with Birth Weight and Gestational Age Among Women in the Second Pregnancy in Eastern Sudan: A Cross-Sectional Study

**DOI:** 10.3390/medicina62101927

**Published:** 2026-10-06

**Authors:** Isameldin E. Medani, Zainab A. Ali, Hatim T. Ali, Nadiah AlHabardi, Ishag Adam

**Affiliations:** 1 Department of Obstetrics and Gynecology, Faculty of Medicine, Jazan University, Jazan 82722, Saudi Arabia; iemedani@jazanu.edu.sa; 2 Faculty of Medicine, Gadarif University, Gadarif 32211, Sudan; 3 Department of Obstetrics and Gynecology, Faculty of Medicine, King Khalid University, Abha 61421, Saudi Arabia; htali@kku.edu.sa; 4 Department of Obstetrics and Gynecology, College of Medicine, Qassim University, Buraydah 52571, Saudi Arabia; n.alhabrdi@qu.edu.sa

**Keywords:** cesarean section, birth weight, gestational age, age, body mass index, Sudan

## Abstract

*Background and Objectives*: The global rate of cesarean sections is on the rise. However, the impact of a prior cesarean section on subsequent birth weight and gestational age remains underexplored. This study aimed to assess the association of a previous cesarean section with the birth weight and gestational age of the current pregnancy among women in their second pregnancy in eastern Sudan. *Materials and Methods*: A cross-sectional study was conducted at Gadarif Maternity Hospital, eastern Sudan, involving 183 women in their second pregnancy. Participants’ sociodemographic and obstetrical data were collected via a questionnaire. Multivariable linear regression analyses were performed. *Results*: The mean (standard deviation) of birth weight and gestational age at delivery were 3030.8 (336.9) grams and 37.7 (1.6) weeks, respectively. Of the total 183, 80 (43.7%) had a previous cesarean section. The prevalences of LBW and preterm birth in the current pregnancy were 35 (19.1%) and 27 (14.8%), respectively. Women with a previous cesarean section had significantly lower mean birth weights (2967.3 g vs. 3080.1 g, *p* = 0.024) and shorter gestational ages (37.3 weeks vs. 38.1 weeks, *p* = 0.001) than women without a previous cesarean section. In multivariable linear regression, only a previous cesarean section was negatively associated with birth weight (coefficient = −102.03, *p* = 0.046). There was no statistical evidence of an association between age, residence, body mass index (BMI), antenatal care (ANC), educational attainment, occupation, and the sex of the newborn and birth weight at delivery. In multivariable linear regression, a previous cesarean section (coefficient = −0.81, *p* < 0.001), BMI (coefficient = −0.07, *p* = 0.022), and the number of ANC visits (coefficient = −0.08, *p* = 0.044) were negatively associated with gestational age. *Conclusions*: For Sudanese women having their second child, a previous cesarean section is associated with lower birth weight and shorter gestational age in the current pregnancy. Healthcare providers should monitor these women to mitigate the potential risks associated with these outcomes.

## 1. Introduction

Cesarean section rates have increased significantly worldwide over the past few decades, as the procedure has evolved from a life-saving intervention to a more commonly performed mode of delivery [1,2]. While a cesarean section can be crucial for maternal and fetal well-being in certain circumstances, the rising prevalence raises concerns about its potential long-term implications for subsequent pregnancies and overall reproductive health [1,2]. Parallel to this trend, the global burden of adverse birth outcomes, specifically low birth weight (LBW) and preterm birth, continues to be a significant public health challenge, as both outcomes contribute significantly to neonatal morbidity and mortality [3,4]. Adverse birth outcomes are much higher among women who live in resource-limited settings, such as Sub-Saharan Africa (SSA) [4,5]. The interrelationship between a previous cesarean section and outcomes in subsequent pregnancies is complex. A prior uterine incision introduces a scar that can influence subsequent placental implantation, uterine integrity, and even fetal growth and development [6]. While studies have explored the risks of uterine rupture and placental (particularly placenta previa and placenta accreta spectrum) disorders and abnormalities in subsequent pregnancies [7,8,9], the direct effect of a previous cesarean section on more subtle fetal growth parameters such as birth weight and gestational age in the subsequent pregnancy remains an area requiring further investigation, particularly in diverse demographic and healthcare settings. Previous studies on how a previous cesarean section affects birth weight (LBW) and gestational age (preterm birth) have shown inconsistent results. Some have shown a negative influence on birth weight [10], gestational age [11,12], or both [8], while others have reported no effect on birth weight [11] or gestational age [13]. However, most of these studies were conducted in high-income countries and non-African contexts, especially those related to the mechanism of the influence [6,7,8,10,13]. There is a noteworthy lack of such research in SSA [11], where cesarean sections and associated complications, such as LBW and preterm birth, are more prevalent [4,5], and no study in Sudan has explored the influence on both birth weight and preterm birth [12]. In low- and middle-income countries, where healthcare resources are often limited and access to comprehensive antenatal care (ANC) varies, understanding these associations is particularly important.

Besides a previous cesarean section, other factors, such as maternal age [14,15], educational attainment [16], area of residence [17], occupation [17], inadequate ANC [18], maternal body mass index (BMI) [14,19], and neonatal sex [19], have been reported to influence neonatal birth weight and gestational age at delivery. The association between birth weight and gestational age is strong [16,19,20], as a lower birth weight may result from preterm birth, intrauterine growth restriction (IUGR), or both [21].

Eastern Sudan, like many regions in SSA, faces unique maternal and child health challenges, including a double burden of communicable and noncommunicable diseases and varying levels of healthcare infrastructure [5,22]. The ongoing war has devastating effects on maternal and perinatal health [23]. Local data on the impact of a previous cesarean section on birth outcomes can inform targeted maternal health interventions and counseling strategies. High rates of cesarean section, LBW, and preterm birth and their associated morbidities and mortalities have been reported in different regions of Sudan, including eastern Sudan [12,24,25,26]. Given the increasing rates of cesarean section and the persistent challenges of LBW and preterm birth globally and in Sudan, a clear understanding of the association between a previous cesarean section and subsequent fetal growth and gestational length is imperative. Thus, this study aimed to contribute to this understanding by assessing the association of a prior cesarean section with birth weight and gestational age in the current pregnancy among women in their second pregnancy in Gadarif, eastern Sudan.

## 2. Materials and Methods

### 2.1. Study Area

Gadarif is one of Sudan’s 18 states. It has about 2.5 million inhabitants [27]. Gadarif City is the capital of Gadarif. Gadarif Maternity Hospital is located in the city center. This government hospital is the state’s main referral hospital. It provides obstetrical and gynecological services to all women regardless of their residency in the state or nearby states.

### 2.2. Study Design and Population

An analytical prospective cross-sectional study was conducted from July to November 2024 at Gadarif Maternity Hospital, Gadarif, eastern Sudan. The authors followed the Strengthening the Reporting of Observational Studies in Epidemiology (STROBE) guidelines [28].

#### Inclusion and Exclusion Criteria

All parturient women in their second pregnancy who attended the hospital for spontaneous delivery (not for induction of labour), regardless of their previous history of cesarean section, were approached to participate after full explanations of the objectives of the study. After signing an informed consent form, all women in their second pregnancy with a singleton pregnancy were enrolled. Primigravida women, multigravida women, women who refused to give informed consent, women with comorbidities such as diabetes mellitus, hypertension, renal disease, or thyroid disease, and women with congenital malformed babies were excluded from the study.

### 2.3. Data Collection and Study Variables

The investigators trained two female medical officers in data-collection methods to standardize the procedure. A questionnaire was developed based on similar studies [8,10,11,12,14,15,16,17,18,19,20]. The questionnaire was designed to collect data on the primary outcome variable (birth weight and gestational age) and independent variables, which are sociodemographic characteristics, including age in years, education level (post-secondary or less), residence (urban or rural), employment status (employed or homemaker), and past obstetrical data, such as a history of cesarean section (yes or no). Data on the index pregnancy, including gravity, number of ANC visits, gestational age, and birth weight at delivery, were collected. Alcohol and smoking are not habits among women in Sudan; hence, they were not included in the questionnaires. Gestational age in weeks was calculated from the last menstrual period and confirmed by an early ultrasound scan. Neonatal birth weight, measured in grams, was recorded immediately after birth. Gestational age and birth weight were categorized as term (≥37 weeks) or preterm (<37 weeks), and as normal birth weight (≥2500 g) or LBW (<2500 g), respectively [29]. The sex of the newborn from the current pregnancy (male or female) was recorded. Additionally, anthropometric measurements, including weight (in kilograms) and height (in centimeters), were taken. The BMI was computed as weight (kg) divided by the square of height (m^2^) [30].

### 2.4. Sample Size Calculation

OpenEpi Menu software Version 3.01 (OpenEpi, Atlanta, GA, USA) was used to compute the desired sample size [31]. A total of 183 women in their second pregnancy were enrolled. A sample calculation of the difference in the means of birth weight and gestational age between women with and without a prior cesarean section was performed using the formula $n=\frac{2{\left(Z_{\alpha /2}+Z_{\beta }\right)}^{2}\sigma^{2}}{d^{2}}$ where *n*: sample size. *Z_α_*_/2_: The critical Z-value for the significance level (α). For a standard 95% confidence level (two-sided α = 0.05), this value is 1.96. *Z_β_*: The critical Z-value for the statistical power (1 − β). For 80% power (β = 0.20), this value is 0.84. σ: The standard deviation. d: The smallest difference between the two group means (μ1 − μ2). This assumption was based on a pilot study that retrospectively (from medical files) compared the birth weight of newborns in women with and without a previous cesarean section (20 women each), reporting lower birth weights of newborns in women with a previous cesarean section than in those without, at 2960 and 3100 g, respectively. Furthermore, based on our earlier study showing a high cesarean section prevalence (43.2%) at a similar referral hospital in central Sudan [25], the authors assumed that 40% of the women had a prior cesarean section. A sample size of 183 (75 women with a previous cesarean section and 108 without) was determined to detect a 5% difference at α = 0.05 with 80% power.

### 2.5. Statistical Analysis

The data were analyzed using IBM Statistical Package for the Social Sciences (SPSS) for Windows (version 22.0; SPSS Inc., New York, NY, USA). Continuous variables, such as age, BMI, gestational age, neonatal birth weight, and number of ANC visits, are expressed as the mean ± standard deviation (SD). A *t*-test was performed to find the difference between the means of neonatal birth weight and gestational age at delivery among women with and without a previous cesarean section. Univariate linear analysis was conducted with neonatal birth weight and gestational age at delivery as the dependent variables, while the independent variables included age, residence, couples’ educational attainment, maternal occupation, BMI, previous cesarean section, ANC, and neonatal sex. Subsequently, variables with *p*-values < 0.20 in the univariate linear analysis were included in a multivariable linear regression, adjusted for covariates. Multicollinearity was checked using the Variance Inflation Factor (VIF), and none was detected (VIF < 4). Model reliability was assessed (R^2^, adjusted R^2^, residual checks, heteroskedasticity, and influential observations). The coefficients, along with their corresponding 95% confidence intervals (CIs), were calculated, and *p*-values < 0.05 were considered statistically significant.

## 3. Results

**Figure 1:**
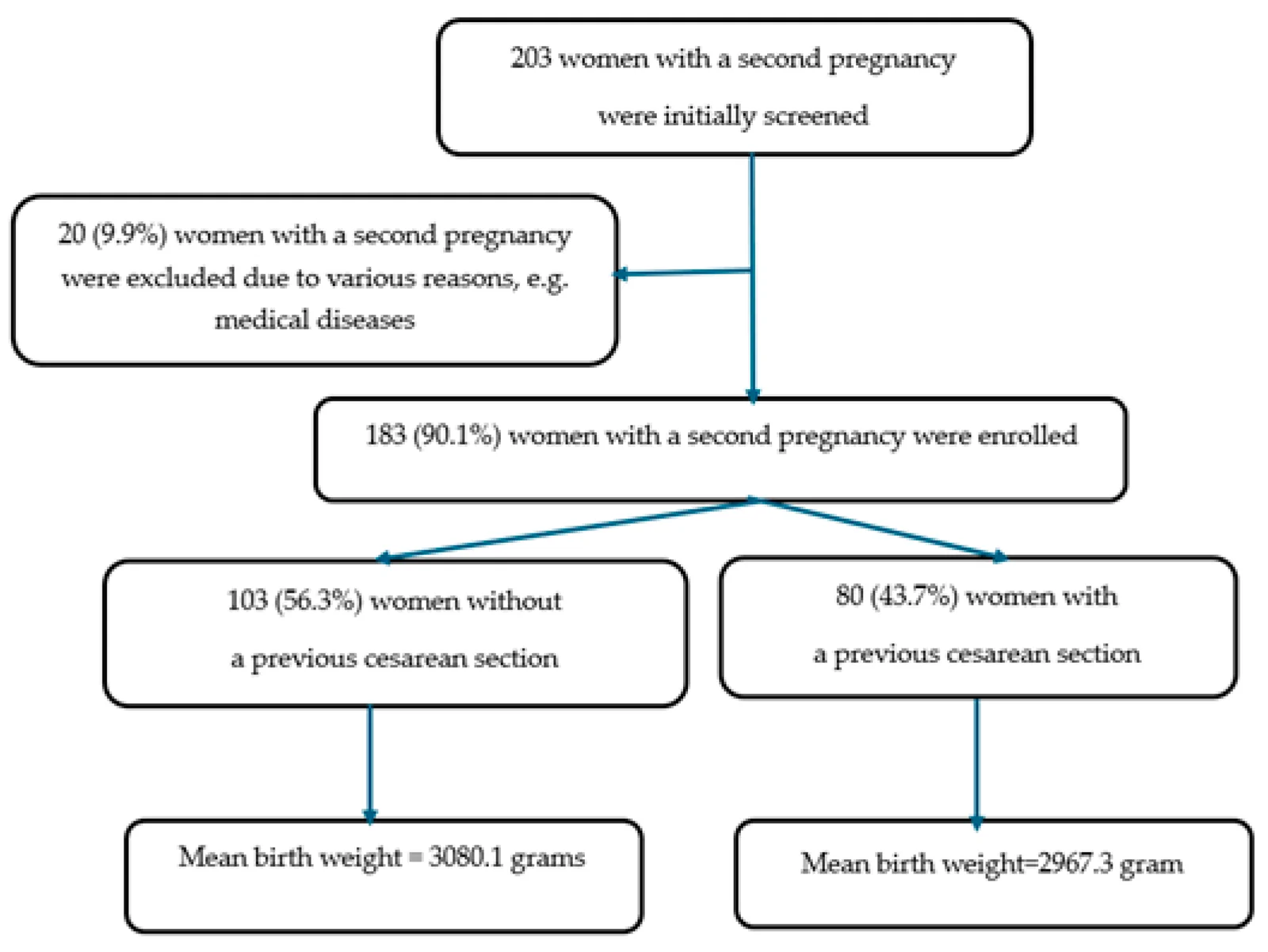
The flow chart of the study of women with a second pregnancy.

Two hundred and three women with a second pregnancy were initially screened. Of these, 20 (9.9%) women with a second pregnancy were excluded for various reasons, e.g., medical diseases. A total of 183 (90.1%) women with a second pregnancy were enrolled (Figure 1).

The mean (SD) age and BMI of the enrolled women were 25.1 (5.3) years and 25.5 (3.6) kg/m^2^, respectively. The mean (SD) of birth weight and gestational age at delivery was 3030.8 (336.9) grams and 37.7 (1.6) weeks, respectively. The mean (SD) number of ANC visits was 5 (2). In all, 115 (62.8%) women were urban residents, while the remaining 68 (37.2%) women were rural residents. A total of 56 (30.6%) women had received a post-secondary education, and 28 (15.3%) were formally employed. A total of 63 (34.4%) of the women’s husbands were educated at a level higher than secondary. Overall, 80 (43.7%) women had a history of previous cesarean section, and the remaining 103 (56.3%) did not. The prevalences of LBW and preterm birth in the current pregnancy were 35 (19.1%) and 27 (14.8%), respectively. In all, 109 (59.6%) gave birth to a male, and the rest, 74 (40.4%), gave birth to a female. There was no difference in age, BMI, race, education, occupation, and sex of newborns between women with and without a previous cesarean section (Table 1).

The *t*-test showed that the mean (SD) birth weight was significantly lower among women with a previous cesarean section than among those without, at 2967.3 (395.7) grams versus 3080.1 (274.9) grams, respectively (*p* = 0.024). The mean (SD) gestational age was significantly lower among women with a history of cesarean section than among those without, at 37.3 (1.4) and 38.1 (1.6) weeks, respectively (*p* = 0.001), Table 1.

In univariate and multivariable linear regression, only a history of previous cesarean section was negatively associated with birth weight at delivery (coefficient −112.80, *p* = 0.024, and coefficient −102.03, *p* = 0.046, respectively) (Table 2 and Table 3). There was no statistical evidence of an association between age, residence, body mass index (BMI), antenatal care (ANC), educational attainment, occupation, and the sex of the newborn and birth weight at delivery. R^2^ = 0.059, adjusted R^2^ = 0.038, mean of residuals = 0.001, Cook’s distance = 0.005.

In univariate linear regression (Table 2), a history of previous cesarean section (coefficient = −0.80, *p* = 0.001), BMI (coefficient = −0.08, *p* = 0.013), and number of ANC visits (coefficient = −0.12, *p* = 0.006) were negatively associated with gestational age at delivery. Giving birth to a female newborn (coefficient = 0.53, *p* = 0.028) was positively associated with gestational age at delivery. Other investigated factors, such as age, area of residence, couples’ education, and maternal occupation, showed no statistical evidence of association with gestational age at delivery.

In multivariable linear regression (Table 3), a history of previous cesarean section (coefficient = −0.81, *p* < 0.001), BMI (coefficient = −0.07, *p* = 0.022), and number of ANC visits (coefficient = −0.08, *p* = 0.044) were negatively associated with gestational age at delivery. Giving birth to a female newborn (coefficient = 0.36, *p* = 0.111) showed no statistical evidence of association with gestational age at delivery (a confounding factor). R^2^ = 0.145, adjusted R^2^ = 0.145, mean of residuals = 0.001, Cook’s distance = 0.012.

## 4. Discussion

This study investigated the association of a prior cesarean section with subsequent birth weight and gestational age among women in their second pregnancy in Gadarif, eastern Sudan. It revealed a negative association between a history of previous cesarean section and both birth weight and gestational age in the current pregnancy. This has important implications for maternal and child health outcomes in a region with unique healthcare challenges. While the average birth weight (3030.8 g) and gestational age (37.7 weeks) fell within typical ranges, the observed prevalences of LBW (19.1%) and preterm birth (14.8%) were concerning. These rates are higher than the global averages reported for low birth weight (LBW) (14.7%) [30] and preterm birth (9.9%) [4], suggesting potential underlying health and socioeconomic vulnerabilities within this population. The high rates of LBW and preterm birth in this study are likely attributable to its government hospital-based design, as referred women may have presented with complications. Residing in an urban area (62.8% of participants) compared to a rural area (37.2% of participants) might also influence access to and the quality of healthcare; however, no association was observed between area of residence and birth weight or gestational age. The key finding of this study was that women with a history of previous cesarean sections delivered neonates with significantly lower mean birth weights (2967.3 g vs. 3080.1 g, *p* = 0.024) and significantly shorter gestational ages (37.3 weeks vs. 38.1 weeks, *p* = 0.001) than those without a prior cesarean section. Specifically, a previous cesarean section was negatively associated with birth weight (coefficient = −102.03, *p* = 0.046) and gestational age (coefficient = −0.81, *p* < 0.001). These findings are consistent with some literature exploring the potential adverse effects of uterine scarring from a previous cesarean section on subsequent pregnancy outcomes [8,10,11,12]. Our last study included 3240 women in central Sudan and revealed that women who had a history of cesarean section were at higher risk for preterm birth [12]. Conditions like placenta previa or placenta accreta, known complications after a cesarean section, can lead to earlier deliveries and necessitate interventions that impact birth weight [6,32]. Placenta accreta itself can lead to neonatal adverse effects (which we did not include in this current study), such as higher neonatal respiratory support requirements [33]. Although the present study did not investigate these specific mechanisms, the observed reductions in birth weight and gestational age may indicate underlying physiological changes. Our results indicate a difference of approximately 102 g in birth weight and nearly one week in gestational age between women with a prior cesarean section and those without. However, the association between a previous cesarean and subsequent birth outcomes is multifaceted. Other studies have reported varied findings, with some observing no significant association or even a positive impact in specific contexts, possibly due to the careful management of pregnancies after cesarean sections [10,11,13]. In northern Tanzania, a retrospective cohort study included 5948 women in their second pregnancy and reported that a prior cesarean was associated with an increased risk of preterm birth but not LBW [11]. In China, Qin et al. reported that while a previous cesarean section negatively affected birth weight by increasing the risk of placenta previa and preterm birth, these adverse effects were mitigated by other factors, such as a higher maternal BMI, which had a positive impact [10]. In the United States, Vahanian et al. reported that a prior cesarean section at term was not associated with an increased risk of low neonatal birth weight or preterm birth in the subsequent pregnancy [13]. Differences in the indication for the previous cesarean section (e.g., failed labor progression vs. fetal distress), the timing of the cesarean section (at term or preterm), the quality of surgical repair, the interpregnancy interval, and the overall standard of ANC could all influence these outcomes.

Our analysis also revealed other factors associated with gestational age. BMI was negatively associated with gestational age (coefficient = −0.07, *p* = 0.022), which might seem counterintuitive given that obesity is often linked to an increased risk of post-term pregnancy [34]. However, maternal obesity is also associated with an increased risk of complications such as preeclampsia and gestational diabetes, which can sometimes necessitate earlier delivery or impact fetal growth [35]. The number of ANC visits also showed a negative association with gestational age (coefficient = −0.08, *p* = 0.044). This finding was unexpected, as increased ANC visits are generally associated with improved maternal and fetal outcomes [36]. This inverse association could suggest reverse causality, where women with concerns about their pregnancy or those experiencing complications might seek more frequent ANC, which could lead to earlier delivery. Alternatively, it might reflect limitations in the quality or impact of ANC in this specific setting [36]. Further qualitative research or more detailed data on the reasons for ANC visits and their content might inform this finding.

While not statistically significant in the multivariable linear regression model (*p* = 0.111), giving birth to a female newborn was positively associated with gestational age in the univariate analysis. Sex-specific differences in fetal growth and maturation are well-documented. Female fetuses often mature earlier and exhibit less susceptibility to growth restriction [37]. Also, the influence of fetal sex on birth weight and gestational age might depend on the presence of prior uterine scarring, particularly that from a cesarean section [38,39]. More research is required to fully understand the complex interplay between fetal sex, placental function, and the presence of uterine scars.

The present study found that birth weight and gestational age were not influenced by several investigated factors, including maternal age, residence, couples’ educational attainment, maternal occupation, or neonatal sex. Likewise, our previous studies determined that these factors were not associated with LBW or preterm birth in Sudan [40,41]. However, as mentioned, some of these factors, such as maternal age [14,15], education [16], residence [17], occupation [17], ANC [18], BMI [14,19], and neonatal sex [19], have been reported to influence neonatal birth weight and gestational age at delivery. These contradictory results in this specific study population in eastern Sudan could be attributed to variations in the measurement of the quality of some variables, such as education. Specifically, education was measured by the number of years of education the women had, rather than the knowledge they acquired. In addition, maternal education was reflected in occupation; relatively few women (15.0%) in this study were formally employed. For example, in African contexts (including the Sudanese context), maternal education had no impact on the prevalence of LBW or preterm birth [41,42]. Researchers should address contradictory findings on how a previous cesarean section influences subsequent pregnancy outcomes, such as birth weight and gestational age, by conducting region-specific studies to establish effective strategies.

### 4.1. Implications

A previous cesarean section was associated with a reduction in birth weight and gestational age in the subsequent pregnancy in eastern Sudanese women. For healthcare providers in eastern Sudan, this means that identifying women with a prior cesarean section is crucial for consideration as a high-risk pregnancy. These women require enhanced ANC focusing and monitoring for signs of preterm labor. Although a previous study did not assess the previous cesarean section, it showed that biochemical markers such as pregnancy-associated plasma protein A (PAPP-A) and free beta-human chorionic gonadotropin (fβ-hCG) could be of help to predict preterm delivery [43]. Public health initiatives should educate women with a prior cesarean section on the potential risks associated with subsequent pregnancies, including lower birth weight and shorter gestational age at delivery, to promote informed decision-making and mitigate these consequences. Furthermore, the findings suggest a need to optimize ANC attendance and address maternal BMI, as these factors also influence gestational age. By addressing these practical implications, healthcare systems can improve maternal and perinatal outcomes in the region.

### 4.2. Strengths and Limitations

This study possesses several notable strengths that enhance its validity and applicability. First, its focus on women in their second pregnancy helped control for parity-related confounding factors, allowing for a more specific examination of the impact of a previous cesarean section. Collecting real-time data reduces reporting bias. Despite its strengths, this study has several limitations that warrant consideration to improve future research. As a cross-sectional study, it can only establish associations and not causality. It cannot determine if the previous cesarean section directly caused lower birth weight or gestational age, or if other, unmeasured factors were at play. While several factors were assessed, unmeasured factors/variables, e.g., family income, may have influenced the relationship between a prior cesarean section and birth outcomes. In addition, no data regarding the types and indications of the previous cesarean section were collected. Although the questionnaire asked the women about the indications for the previous cesarean section, the majority did not know them. Knowing these factors, such as whether the cesarean section was elective or emergency, and its indication, would have provided a more comprehensive understanding of the observed associations. Birthweight centiles or z-scores adjusted for gestational age and sex were not assessed. Finally, as the study was conducted in eastern Sudan, the findings might not be directly generalizable to populations with different socioeconomic, healthcare, or genetic backgrounds.

## 5. Conclusions

This study in eastern Sudan showed that a history of previous cesarean section is associated with lower birth weight and shorter gestational age in the pregnancy of women in their second pregnancy. However, these findings do not challenge the indications for cesarean delivery when medically necessary. Future longitudinal studies with larger sample sizes and more detailed clinical data are warranted to explore further the mechanisms underlying these associations and to develop targeted interventions to improve birth outcomes in this vulnerable population.

## Figures and Tables

**Table 1:** General characteristics of the studied women in their second pregnancy with and without previous cesarean section in eastern Sudan, 2024.

		All Women (Number = 183)	Women with a Previous Cesarean Section (Number = 80)	Women Without a Previous Cesarean Section (Number = 103)	*p* Value
Variable		Mean (standard deviation) of			
Age, years		25.1 (5.3)	25.5 (5.4)	24.8 (5.2)	0.339
Body mass index, kg/m^2^		25.5 (3.6)	25.2 (3.9)	25.7 (3.3)	0.364
Birth weight at delivery, grams		3030.8 (336.9)	2967.3 (395.7)	3080.1 (274.9)	0.024
Gestational age at delivery, weeks		37.7 (1.6)	37.3 (1.4)	38.1 (1.6)	0.001
		Frequency (Percentage) of			
Residence	Urban	115 (62.8)	46 (57.5)	69 (67.0)	0.218
	Rural	68 (37.2)	34 (42.4)	34 (33.0)	
Maternal education level	>Secondary	56 (30.6)	21 (26.3)	35 (34.0)	0.332
	≤Secondary	127 (69.4)	59 (73.8)	68 (66.0)	
Husband’s education level	>Secondary	63 (34.4)	22 (27.5)	41 (39.8)	0.087
	≤Secondary	120 (65.6)	58 (72.5)	62 (60.2)	
Maternal occupational status	Housewives	155 (84.7)	70 (87.5)	85 (82.5)	0.411
	Employed	28 (15.3)	10 (12.5)	18 (17.5)	
Preterm birth	No	156 (85.2)	62 (77.5)	94 (91.3)	0.012
	Yes	27 (14.8)	18 (22.5)	9 (8.7)	
Low birth weight	No	148 (80.9)	61 (76.3)	87 (84.5)	0.187
	Yes	35 (19.1)	19 (23.8)	16 (15.5)	
Sex of newborn	Male	109 (59.6)	49 (61.3)	60 (58.3)	0.762
	Female	74 (40.4)	31 (38.8)	43 (41.7)	

**Table 2:** Univariate linear analysis of factors associated with birth weight and gestational age at delivery in women in their second pregnancy in eastern Sudan (N = 183), 2024.

Variable		Birth Weight at Delivery, Grams			Gestational Age at Delivery, Weeks		
		Coefficient	95.0% CI	*p*-Value	Coefficient	95.0% CI	*p*-Value
Age, years		−2.39	−11.65–6.86	0.610	0.01	−0.02–0.05	0.503
Body mass index, kg/m^2^		1.58	−12.14–15.30	0.820	−0.08	−0.14 to −0.01	0.013
Number of antenatal care visits		18.40	−0.21–37.01	0.053	−0.12	−0.21 to −0.03	0.006
Residence	Urban	Reference					
	Rural	−78.80	−180.12–22.51	0.127	−0.130	−0.61–0.35	0.598
Maternal education level	>Secondary	Reference					
	≤Secondary	−23.72	−130.60– 83.15	0.662	−0.20	−0.71–0.31	0.442
Husband’s education level	>Secondary	Reference					
	≤Secondary	−4.17	−107.88–99.54	0.937	−0.17	−0.67–0.31	0.482
Maternal occupational status	Homemaker	Reference					
	Employed	100.79	−35.28–236.88	0.146	0.27	−0.37–0.92	0.407
Previous cesarean section	No	Reference					
	Yes	−112.80	−210.76 to −14.84	0.024	−0.80	−1.26 to −0.34	0.001
Sex of newborn	Male	Reference					
	Female	−45.90	−146.09–54.27	0.367	0.53	0.05–1.01	0.028

**Table 3:** Multivariable linear regression of factors associated with birth weight and gestational age at delivery in women in their second pregnancy in eastern Sudan (N = 183), 2024.

Variable		Birth Weight at Delivery, Grams			Gestational Age at Delivery, Weeks		
		Coefficient	95.0% CI	*p*-Value	Coefficient	95.0% CI	*p*-Value
Body mass index, kg/m^2^		–	–	–	−0.07	−0.13 to −0.01	0.022
Number of antenatal care visits		16.96	−1.88–35.81	0.077	−0.08	−0.17 to −0.002	0.044
Residence	Urban	Reference					
	Rural	−39.63	−148.05–68.78	0.472	–	–	–
Maternal occupational status	Homemaker	Reference					
	Employed	47.19	−94.19–188.57	0.511	–	–	–
Previous cesarean section	No	Reference					
	Yes	−102.03	−202.25 to −1.80	0.046	−0.81	−1.26 to −0.37	<0.001
Sex of newborn	Male	Reference					
	Female	–	–	–	0.36	−0.08–0.82	0.111

## Data Availability

The data supporting the current study’s findings are available from the corresponding author upon reasonable request.
